# The Refeeding Syndrome: a neglected but potentially serious condition for inpatients. A narrative review

**DOI:** 10.1007/s11739-020-02525-7

**Published:** 2020-10-19

**Authors:** Valentina Ponzo, Marianna Pellegrini, Iolanda Cioffi, Luca Scaglione, Simona Bo

**Affiliations:** 1 Department of Medical Sciences, University of Torino, c.so AM Dogliotti 14, 10126 Turin, Italy; 2 Department of Clinical Medicine and Surgery, Federico II University Hospital, Naples, Italy; 3 Internal Medicine Unit, Città della Salute e della Scienza Hospital of Torino, Turin, Italy

**Keywords:** Hypophosphatemia, Hypokalemia, Hypomagnesemia, Malnutrition, Refeeding syndrome, Thiamine

## Abstract

The Refeeding Syndrome (RFS) is a potentially serious, but still overlooked condition, occurring in individuals who are rapidly fed after a period of severe undernourishment. RFS derives from an abnormal electrolyte and fluid shifts leading to many organ dysfunctions. Symptoms generally appear within 2–5 days of re-feeding and may be absent/mild or severe and life threating, depending on the pre-existing degree of malnutrition and comorbidities. The lack of a standard definition and the nonspecificity of the symptoms make both incidence estimate and diagnosis difficult. In 2020, the American Society for Parenteral and Enteral Nutrition (ASPEN) proposed a unifying definition for the RFS and its severity classification. The awareness of the condition is crucial for identifying patients at risk, preventing its occurrence, and improving the management. The objectives of this narrative review were to summarize the current knowledge and recommendations about the RFS and to provide useful tips to help physicians to recognize and prevent the syndrome.

**Electronic supplementary material:**

The online version of this article (10.1007/s11739-020-02525-7) contains supplementary material, which is available to authorized users.

## Introduction

Malnutrition is a frequent and often unrecognized condition among inpatients [1, 2]. Indeed, 20–50% of individuals are at risk of malnutrition or already malnourished at hospital admission, but malnutrition is diagnosed in 7% only [3]. Older age, low socioeconomic status, lack of organizational support, chronic systemic or psychiatric diseases, polytherapy, poor diet, reduced absorption capacity, excessive nutrient losses are the most frequent conditions underlying malnutrition [4]. The management of malnourished inpatients can be difficult due to the risk of metabolic impairment after the start of nutrition [5]. The adverse outcomes of refeeding were firstly reported during the World War II in rapidly re-fed prisoners who had starved for five to six months [6]. People who have fasted for a long time, developed heart, and/or respiratory failure, peripheral edema, neurological symptoms, and death after the introduction of excessive or even appropriate calorie amount [6–8]. In the 80 s, the term ‘refeeding syndrome’ (RFS) was introduced to describe severe hypophosphatemia and other electrolyte/metabolic abnormalities and the related cardiovascular and pulmonary manifestations leading to death occurring in two chronically malnourished patients who received aggressive dextrose‐based parenteral nutrition (PN) [9]. Since then, many cases of RFS have been described as a rare, but severe and potentially fatal complication related to re-feeding (either orally, enterally or parenterally) of individuals who have fasted or consumed very few calories over a long period of time [10, 11]. Among the diseases or conditions predisposing to malnutrition and consequently to RFS after re-feeding, anorexia nervosa [12–14], cancer [15, 16], critical illnesses [13, 17–20], and frailty in the elderly [21–27] are the most frequently implicated.

The switch from a catabolic to an anabolic state may be the cause of the clinical manifestations of the RFS, even though the pathophysiological mechanisms are still not fully understood [28]. Furthermore, the lack of a clear definition accounts for the difficulty of diagnosis and uncertainties in treatment [2, 29]. Therefore, the RFS is a potentially serious condition, often overlooked by many physicians [30]. This is of particular concern because of the high prevalence of hospital malnutrition often underestimated even in the internal medicine wards [31, 32].

The objectives of this narrative review are to summarize the knowledge on the RFS and to focus on the most useful topics for the clinical practice.

## Methods

The following databases were queried: PubMed (National Library of Medicine), the Cochrane Library, Excerpta Medica dataBASE (EMBASE), and the Cumulative Index to Nursing and Allied Health Literature (CINAHL). The search strategy was performed using the following keywords: refeeding syndrome OR phosphate, potassium, magnesium AND anorexia nervosa, cancer, critically ill patients, elderly. The filters ‘humans’ and ‘adults’ were used. Hand searching the references of the identified studies and reviews was carried out too.

## Incidence rates for RFS

The lack of a universally recognized RFS definition makes it difficult to obtain precise estimates of its incidence [33]. Indeed, either hypophosphatemia only or multiple electrolyte abnormalities (with different cut offs) with or without clinical manifestations have been considered in its definition [34, 35]. The reported incidence rates ranged between 0 and 80%, depending on the definition and the patient population studied [34]. RFS has been described in 48% of severely malnourished patients, in 34% of intensive care unit (ICU) patients, in 33% of patients with anorexia nervosa (AN), in 25% of cancer inpatients, and in 9.5% of patients hospitalized for malnutrition from gastrointestinal fistulae [10, 12, 33, 36]. Many factors may lead to underestimation of RFS incidence rate, such as insufficient monitoring of the patients’ electrolytes after nutrition starting, lack of consultation by experts in clinical nutrition, the nonspecificity of the clinical manifestations of the syndrome in patients with multiple co-morbidity and the physician unawareness [11].

## Population at risk for RFS

To identify patients at risk for RFS is necessary evaluating the risk of malnutrition by validated screening tools first, and then assessing the diagnosis and grading the severity of malnutrition [5, 33, 37, 38]. Distinguishing malnutrition from the other related conditions, such as starvation, cachexia, cancer cachexia, and sarcopenia, is important from a clinical point of view (Table 1) [39–44]. The screening for the risk of malnutrition should be performed in inpatients within the first 24–48 h through validated screening tools, such as the Nutritional Risk Screening 2002 (NRS-2002), the Mini Nutritional Assessment-Short Form (MNA-SF), the Malnutrition Universal Screening Tool (MUST), the Short Nutritional Assessment Questionnaire (SNAQ) [5, 37, 39]. If an individual is identified to be at risk of malnutrition, an extensive nutritional assessment for diagnosis and evaluation of the severity of malnutrition should be carried out by an expert in nutrition [39, 40].

**Table 1:** Definition of malnutrition and other related conditions

Malnutrition [40]
At least 1 phenotypic criterion and 1 etiologic criterion should be present Phenotypic Criteria: Nonvolitional weight loss Low body mass index Reduced muscle mass Etiologic criteria: Reduced food intake or assimilation Disease burden/inflammation condition
Starvation [44]
Reduction in both fat and fat-free mass due to protein–energy deficiency, which could be reversed solely by the provision of nutrients
Cachexia [42]
Severe weight loss (adults) or growth failure (children) due to loss of muscle ± loss of fat mass associated with increased protein catabolism by underlying chronic illness
Cancer cachexia [41]
A multifactorial syndrome defined by an ongoing loss of skeletal muscle mass (with or without loss of fat mass) that cannot be fully reversed by conventional nutritional support and leads to progressive functional impairment
Sarcopenia [43]
Sarcopenia is a progressive and generalized skeletal muscle disorder that is associated with increased likelihood of adverse outcomes including falls, fractures, physical disability, and mortality.
Sarcopenia is *probable* when low muscle strength is detected (handgrip strength < 27 kg for males and < 16 kg for females). A sarcopenia diagnosis is *confirmed* by the presence of low muscle quantity or quality (ASM/height^2^ < 7.0 kg/m^2^ for males and < 5.5 kg/m^2^ for females). When low muscle strength, low muscle quantity/quality and low physical performance (low gait speed ≤ 0.8 m/s both for males and females) are all detected, sarcopenia is considered severe

*BMI* body mass index, *ASM* appendicular skeletal muscle mass

A great number of diseases or conditions predisposes to malnutrition [21, 28, 33, 34, 37, 39, 45–47]. These predisposing conditions can be divided into the following categories: predisposing to disease-related malnutrition with inflammation (chronic diseases leading to catabolic inflammatory responses); predisposing to disease-related malnutrition without inflammation (acute disease and injury-related malnutrition); and predisposing to malnutrition in the absence of diseases (hunger, socioeconomic, or psychologic-related conditions, drugs) [39], as summarized in Supplementary Table 1.

In the presence of severe underweight or weight loss, prolonged fasting period, and/or low electrolyte concentrations, the risk of RFS is particularly high [30]. In 2006, the National Institute for Health and Clinical Excellence (NICE) guidelines [48] reported the risk factors to identify people at low or high risk for RSF. In 2018 Friedli et Coll added the very high-risk category [21]. Recently, the American Society for Parenteral and Enteral Nutrition (ASPEN) published updated consensus criteria for identifying adult patients at risk for RFS [33]. These criteria are presented in Table 2.

**Table 2:** Criteria for identifying adult patients at risk for RFS

	NICE [48]		ASPEN 2020 [33]		Friedli 2018 [21]		
	High risk in the presence of		Moderate risk: 2 risk criteria needed	Significant risk: 1 risk criteria needed	Low risk: 1 minor risk factor	High risk: 1 major or 2 minor risk factors	Very high risk
	One or more of the following:	Two or more of the following:			Minor risk factors	Major risk factors	
BMI	< 16 kg/m^2^	< 18.5 kg/m^2^	16–18.5 kg/m^2^	< 16 kg/m^2^	< 18.5 kg/m^2^	< 16 kg/m^2^	< 14 kg/m^2^
Weight loss	> 15% within the last 3–6 months	> 10% within the last 3–6 months	5% in 1 month	7.5% in 3 months or > 10% in 6 months	> 10% within the last 3–6 months	> 15% within the last 3–6 months	> 20%
Caloric intake	Little or no nutritional intake > 10 days	Little or no nutritional intake > 5 days	None or negligible oral intake for 5–6 days OR < 75% of estimated energy requirement for > 7 days during an acute illness or injury OR < 75% of estimated energy requirement for > 1 month	None or negligible oral intake for > 7 days OR < 50% of estimated energy requirement for > 5 days during an acute illness or injury OR < 50% of estimated energy requirement for > 1 month	Little or no nutritional intake > 5 days	Little or no nutritional intake > 10 days	Starvation > 15 days
Prefeeding potassium, phosphate, or magnesium serum concentrations	Low levels		Minimally low levels or normal current levels and recent low levels necessitating minimal or single‐dose supplementation	Moderately/significantly low levels or minimally low or normal levels and recent low levels necessitating significant or multiple‐dose supplementation		Low levels	
Loss of subcutaneous fat			Evidence of moderate loss	Evidence of severe loss			
Loss of muscle mass			Evidence of mild or moderate loss	Evidence of severe loss			
Higher‐risk comorbidities*		A history of alcohol abuse or drugs including insulin, chemotherapy, antacids, or diuretics	Moderate disease	Severe disease		A history of alcohol abuse or drugs including insulin, chemotherapy, antacids, or diuretics	

*BMI* body mass index

^*^Acquired immunodeficiency syndrome; Advanced neurologic impairment or general inability to communicate needs; Cancer; Chronic alcohol or drug use disorder; Dysphagia and esophageal dysmotility; Eating disorders; Food insecurity and homelessness; Failure to thrive, including physical and sexual abuse and victims of neglect; Hyperemesis gravidarum or protracted vomiting; Major stressors or surgery without nutrition for prolonged periods of time; Malabsorptive states (e.g., short‐bowel syndrome, Crohn’s disease, cystic fibrosis, pyloric stenosis, maldigestion, pancreatic insufficiency); Postbariatric surgery; Postoperative patients with complications; Prolonged fasting; Protein malnourishment; Refugees

## Diagnosis of RFS

The difficulty in RFS diagnosing is due to the discrepancy between the onset of the symptoms and the occurring of metabolic shift (see below), and the nonspecific nature of its clinical manifestations [46]. There is a great heterogeneity among the published definitions of RFS, ranging from hypophosphatemia alone [18, 19, 22, 24, 27, 49–54] to the presence of severe low-serum electrolyte levels along with fluid balance abnormalities and/or organ dysfunction [16, 21, 34, 55]. Only hypophosphatemia has been universally recognized as a feature of the syndrome [38]. Friedli et Coll. proposed diagnostic criteria for imminent or manifest RFS, based on the electrolyte blood concentrations and clinical symptoms to standardize its prevention and treatment [21]. According to this definition, “imminent” RFS is present when a shift in electrolytes occurs within 72 h after the start of nutritional treatment (i.e., > 30% decrease in blood phosphate from baseline or phosphate values < 0.6 mmol/L or any two other electrolyte shifts below normal range) [21]. “Manifest” RFS is considered if any electrolyte shift occurs in conjunction with typical clinical symptoms (see below) [21].

More recently, the ASPEN proposed diagnostic criteria for distinguishing mild, moderate or severe RFS [33] (Table 3). The extent of the decrease in the serum levels of one or more electrolytes (among phosphate, potassium, or magnesium) defines RFS severity: 10–20% (mild RFS), 20–30% (moderate RFS), > 30% and/or organ dysfunction and/or thiamine deficiency (severe RFS) [33]. Thus, either hypophosphatemia and/or hypokalemia and/or hypomagnesemia qualify the presence of the RFS. The timing of onset is determinant for the diagnosis, since the RFS develops shortly (from hours up to 5 days) after having substantially increased the energy provision to individuals who have been undernourished [33].

**Table 3:** Diagnostic criteria for RFS severity [33]

Severity of RFS	Mild	Moderate	Severe
Serum electrolytes*	10–20% less	20–30% less	> 30% less and/or organ dysfunction**
Timing	From hours up to 5 days after increasing the energy provision in an individual at risk		

*Decrease in any (one or more) of electrolyte serum levels, among phosphate, potassium, and/or magnesium

**Resulting from the decrease in any electrolyte and/or from thiamine deficiency

## Pathophysiology and clinical manifestations

The pathophysiology of the RFS is probably related to the shift from the catabolic to the anabolic metabolic pathways occurring after the re-start of feeding in undernourished subjects. During early starvation, blood glucose and insulin levels decline while glucagon concentrations increase by stimulating glycogenolysis in the liver. When glycogen reserves become depleted, gluconeogenesis is stimulated in the liver, using amino acids derived from muscle breakdown [56]. During prolonged fasting, the body switches to use fats as the main sources of energy with a decrease in basal metabolic rate of 20–25% [57]. Increased lipolysis in fat reserves leads to the production of ketones that are used by the brain as preferred fuel during starvation [29, 56]. During prolonged fasting, several intracellular minerals become severely depleted, particularly phosphate, potassium, and magnesium. However, the concentrations of these minerals may remain within the normal range in the serum because there is a reduction in their renal excretion and because of the phosphate outflow from the cells into the blood, leading to normal blood phosphate levels despite depleted storages [21].

Symptoms generally appear within 2–5 days of re-feeding and may range from absent/mild to a severe and life-threating clinical syndrome, depending on the pre-existing degree of malnutrition and comorbidity [10, 11, 45]. All the body organs may be involved, leading to cardiac, respiratory, hematologic, gastrointestinal, neurologic, and musculoskeletal manifestations, until death [10, 21, 58].

### Insulin and carbohydrate metabolism

Rapid refeeding in a starved patient causes the metabolic and hormonal changes underlying the syndrome [59]. The provision of nutrients, above all carbohydrates, increases insulin secretion and promotes a sudden shift from fat to carbohydrates metabolism. Insulin stimulates the sodium potassium ATPase symporter, with magnesium as co-factor, which transports glucose and potassium into the cells and moves out sodium. Moreover, insulin release stimulates anabolic processes that require minerals (promoting cellular uptake of phosphate, potassium, and magnesium) and coenzymes, such as thiamine [29]. The electrolyte shift, along with the depletion of the mineral pool, could lead to profound hypophosphatemia and low extracellular magnesium and potassium concentrations, but not necessarily to the depletion of all together. Furthermore, insulin has an anti-natriuretic effect on renal tubules causing a decrease in urinary sodium and water excretion [59]. This determines a rapid fluid overload that can lead to congestive cardiac failure, arrhythmia, and pulmonary edema.

### Hypophosphatemia

The phosphate is predominantly an intracellular mineral that plays a key role in energy production and transfer (as a component of adenosine triphosphate (ATP) [58] and it is necessary for many enzymatic processes of cellular metabolic pathways [60]. During refeeding, the increased phosphate consumption due to enhanced production of phosphorylated intermediates results in reduced generation of ATP and 2,3-diphosphoglycerate with impaired cardiac and respiratory functions, and decreased oxygen release to the tissues (Table 4).

**Table 4:** Physiopathology and main clinical features of the RFS

Pathophysiological mechanisms	Clinical manifestations
Hypophosphatemia	
Increased phosphate consumption due to enhanced production of phosphorylated intermediates for glycolysis, the Krebs cycle, and the electron transport chain to produce adenosine triphosphate and 2,3-diphosphoglycerate	Impaired cardiac and respiratory functions (i.e., tachycardia and tachypnea)
	Neurologic symptoms (i.e., confusion, somnolence, lethargy, coma, paresthesia, seizures)
	Hematologic disorders (i.e., hemolysis, dysfunction of platelets and leukocytes, thrombocytopenia)
	Hypoxia (due to impaired oxygen release from 2,3- diphosphoglycerate)
	Muscular disorders (i.e., weakness, rhabdomyolysis, decreased cardiac contractility, myalgia)
Hypokalemia	
Intracellular shift of potassium by insulin stimulation of the Na + /K + ATPase	Cardiac arrhythmias
Impairment of potassium reuptake in the nephron (role of hypomagnesemia)	Neurologic symptoms (i.e., weakness, hyporeflexia, respiratory depression, and paralysis) due to impaired transmission of electrical impulses
Hypomagnesemia	
Not completely clear Intracellular shift of magnesium after carbohydrate feeding	Increased renal losses of potassium
	Cardiac arrhythmias (i.e., torsade de pointes, atrial fibrillation, ventricular arrhythmias)
	Electrocardiograph changes (i.e., prolonged QT and PR, widened QRS)
	Abdominal discomfort (i.e., anorexia, diarrhea, nausea, vomiting)
	Neuromuscular symptoms (i.e., tremor, paraesthesia, tetany, seizures, irritability, confusion, weakness, ataxia)
Thiamine deficiency	
Increased consumption of thiamine by glucose metabolism enzymes	Neurologic disorders or dry beriberi, Wernicke encephalopathy and Korsakoff’s syndrome (i.e., ataxia, disturbance of consciousness, oculomotor abnormalities, symptoms of acute peripheral neuropathy, coma)
	Cardiovascular disorders or wet beriberi (i.e., peripheral edema, heart failure)
	Metabolic acidosis (due to glucose conversion to lactate)
Sodium and fluid retention	
Renal sodium and fluid retention due to insulin antinatriuretic properties (after carbohydrate feeding)	Peripheral edema
	Pulmonary edema and heart failure (due to increased vasoconstriction and peripheral resistance by sodium stimulation of noradrenaline and angiotensin II)
Hyperglycemia	
Increased tissue resistance to endogenous glucose	Metabolic acidosis
	Hypercapnia, respiratory failure, and risk of fatty liver due to lipogenesis (stimulated by insulin)

*ATP* adenosine triphosphate

### Hypokalemia

Potassium is an intracellular mineral and it is crucial for the maintenance of the sodium–potassium membrane gradient; hypokalemia causes imbalance in the electrochemical membrane potential and impaired transmission of electrical impulses resulting in arrhythmias, cardiac arrest, and neurologic symptoms [61–63].

### Hypomagnesemia

Magnesium plays a role as a cofactor for the phosphorylation of ATP and it is important for the maintenance of neuromuscular and enzymatic functions. Its depletion results in increased renal losses of potassium, aggravating hypokalemia with arrhythmias and ECG abnormalities, and in abdominal discomfort and neuromuscular symptoms [64].

### Thiamine deficit

Thiamine is another cofactor in ATP production. Its increased consumption during refeeding by the enhanced activity of enzymes implicated in the carbohydrate metabolism may lead to neurologic disorders (dry beriberi, Wernicke encephalopathy and Korsakoff’s syndrome), cardiovascular disorders, and metabolic acidosis (due to the conversion of glucose into lactate) [65] (Table 4).

## Prevention and treatment

The identification of patients at risk for RFS is the first step to prevent the onset of the syndrome, and to avoid an excessive nutritional replenishment in those individuals [21, 66]. Risk factors should be carefully investigated before starting either oral, enteral, or parenteral nutrition, because every route of calorie administration is implicated in the occurrence of the RFS [33, 58]. Well-trained medical staff and specialized nutritional support teams, consisting of physicians, dieticians, nurses, and pharmacists, positively impact on the patient outcomes [48]. However, a multidisciplinary team is not available in all hospital settings, and often the evaluation of the risk for RFS is left to the clinician's critical sense at the time of starting nutritional support [11, 33, 36, 38, 67]. After defining the degree of RFS risk, the rate of fluid and nutrition administration, the correction of electrolyte imbalances, and the supplementation of vitamins and micronutrients (zinc, iron, selenium) can be determined [36] (Table 5). If a prolonged nutritional support is required, adjustments over time in accordance with the patient clinical conditions might be necessary [58].

**Table 5:** Prevention and treatment of the RFS according to the risk [21, 36, 38]

Day	Treatment	Low risk	High risk	Very high risk	Monitoring
1	Thiamine	200–300 mg	200–300 mg	200–300 mg	Body weight Vital signs Clin Exam Lab tests§
	Multivitamin*	Yes	Yes	Yes	
	Sodium restriction	No	< 1 mmol/kg/day	< 1 mmol/kg/day	
	Fluids	30–35 ml/kg/day	25–30 ml/kg/day	20–25 ml/kg/day	
	Nutritional support**	15–25 kcal/kg/day	10–15 kcal/kg/day	5–10 kcal/kg/day	
2	Thiamine	200–300 mg	200–300 mg	200–300 mg	Body weight Vital signs Clin Exam Lab tests§
	Multivitamin*	Yes	Yes	Yes	
	Sodium restriction	No	< 1 mmol/kg/day	< 1 mmol/kg/day	
	Fluids	30–35 ml/kg/day	25–30 ml/kg/day	20–25 ml/kg/day	
	Nutritional support**	15–25 kcal/kg/day	10–15 kcal/kg/day	5–10 kcal/kg/day	
3	Thiamine	200–300 mg	200–300 mg	200–300 mg	Body weight Vital signs Clin Exam Lab tests§
	Multivitamin*	Yes	Yes	Yes	
	Sodium restriction	No	< 1 mmol/kg/day	< 1 mmol/kg/day	
	Fluids	30–35 ml/kg/day	25–30 ml/kg/day	20–25 ml/kg/day	
	Nutritional support**	15–25 kcal/kg/day	10–15 kcal/kg/day	5–10 kcal/kg/day	
4	Thiamine	No	No	200–300 mg	Vital signs Clin Exam
	Multivitamin*	Yes	Yes	Yes	
	Sodium restriction	No	< 1 mmol/kg/day	< 1 mmol/kg/day	
	Fluids	30–35 ml/kg/day	30–35 ml/kg/day	25–30 ml/kg/day	
	Nutritional support**	30 kcal/kg/day	15–25 kcal/kg/day	10–20 kcal/kg/day	
5	Thiamine	No	No	200–300 mg	Body weight Vital signs Clin Exam Lab tests§
	Multivitamin*	Yes	Yes	Yes	
	Sodium restriction	No	< 1 mmol/kg/day	< 1 mmol/kg/day	
	Fluids	30–35 ml/kg/day	30–35 ml/kg/day	25–30 ml/kg/day	
	Nutritional support**	full requirements	15–25 kcal/kg/day	10–20 kcal/kg/day	
6	Multivitamin*	Yes	Yes	Yes	Vital signs Clin Exam
	Sodium restriction	No	< 1 mmol/kg/day	< 1 mmol/kg/day	
	Fluids	30–35 ml/kg/day	30–35 ml/kg/day	25–30 ml/kg/day	
	Nutritional support**	full requirements	25–30 kcal/kg/day	10–20 kcal/kg/day	
7	Multivitamin*	Yes	Yes	Yes	Vital signs Clin Exam
	Sodium restriction	No	< 1 mmol/kg/day	< 1 mmol/kg/day	
	Fluids	30–35 ml/kg/day	30–35 ml/kg/day	30–35 ml/kg/day	
	Nutritional support**	full requirements	full requirements	20–30 kcal/kg/day	
8	Multivitamin*	Yes	Yes	Yes	Vital signs Clin Exam
	Sodium restriction	No	No	< 1 mmol/kg/day	
	Fluids	30–35 ml/kg/day	30–35 ml/kg/day	30–35 ml/kg/day	
	Nutritional support**	full requirements	full requirements	20–30 kcal/kg/day	
9	Multivitamin*	Yes	Yes	Yes	Body weight Vital signs Clin Exam Lab tests§
	Sodium restriction	No	No	< 1 mmol/kg/day	
	Fluids	30–35 ml/kg/day	30–35 ml/kg/day	30–35 ml/kg/day	
	Nutritional support**	Full requirements	Full requirements	20–30 kcal/kg/day	
10	Multivitamin*	Yes	Yes	Yes	Vital signs Clin Exam
	Sodium restriction	No	No	< 1 mmol/kg/day	
	Fluids	30–35 ml/kg/day	30–35 ml/kg/day	30–35 ml/kg/day	
	Nutritional support**	Full requirements	Full requirements	Full requirements	

*Clin Exam* clinical examination

*Vitamins should be supplemented to 200% and the trace elements to 100% of the recommended daily intakes; replace electrolyte according to the electrolyte serum levels and RFS severity: 1–1.5 mmol/Kg/day potassium, 0.2–0.4 mmol/Kg/day magnesium, 0.3–0.6 mmol/Kg/day phosphate

**Provide 15–20% proteins, 30–40% carbohydrates, 40–60% fats

§Laboratory tests include phosphate, sodium, potassium, magnesium, calcium, glucose, creatinine, urea

**Fig. 1:**
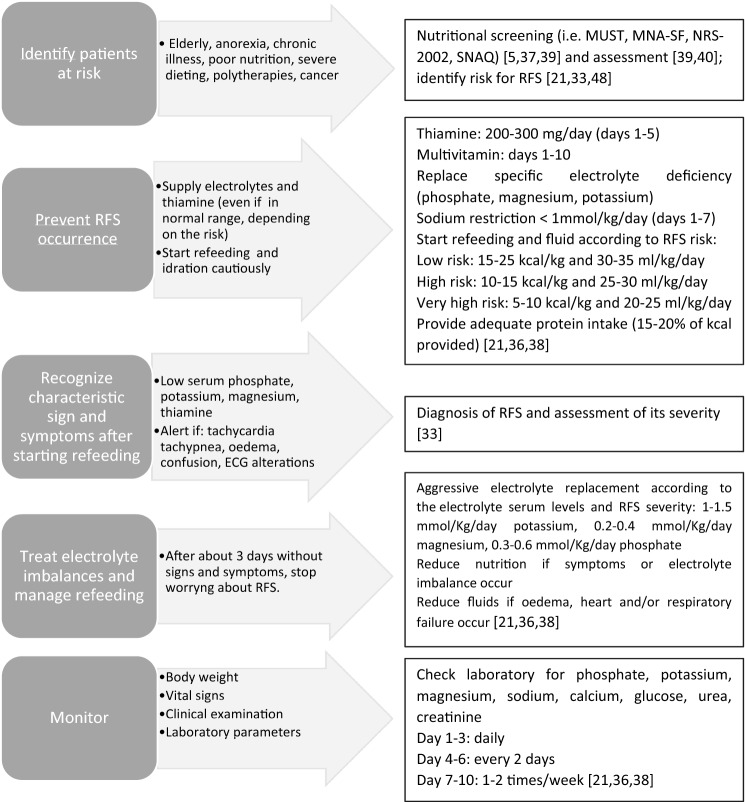
Practical tips for the prevention and approach to the RFS

Several therapeutic approaches have been proposed to prevent or treat the RSF [10, 21, 28, 36, 45, 46, 48, 67, 68] (Fig. 1). Since hypophosphatemia occurs after refeeding, according to the grade of RSF risk, phosphate may be administered preventively before the initiation of nutritional therapy, even if blood levels are in the low-normal range [21]. Similarly, thiamine is essential in carbohydrates metabolism and should be supplemented before restart feeding even in the case of normal blood levels [21]. An excessive administration of glucose by stimulating insulin production leads to the consumption of electrolytes (mainly phosphate) through the anabolic pathways. Starting re-feeding very gradually, independently of the route of administration, is therefore mandatory [58]. Owing to the risk of fluid overload, sodium and hydration should be provided cautiously, until the patient is metabolically stable [38]. In case of overt symptoms, energy and fluid intakes should be reduced and adapted to the clinical conditions [30].

Specific conditions might require special attention.

### Anorexia nervosa

Most inpatients with AN are at high risk for RFS [12]; refeeding is the first step of the treatment and must be managed very cautiously [66, 69]. International guidelines are based mainly on clinical experience, due to the lack of well-designed trials in inpatients with AN [70, 71]. At hospital admission, the recommended calorie provision ranges from 5–20 kcal/kg to 30–40 kcal/kg [70, 71]. A progressive increase of 5–10 kcal/kg/day (if high risk of RFS) or 10–20 kcal/kg/day (if moderate risk of RFS) could be carried out after the stabilization of the clinical conditions (e.g., improvement of electrocardiographic abnormalities, correction of electrolyte imbalance, replacement of thiamine and vitamins, and stabilization of comorbidities) [48, 66, 72]. Caloric provision could increase up to 70–100 kcal/kg per day if patients have increased energy requirement such as in case of inappropriate behaviors (throwing or hiding food, vomiting, intense exercise, etc.) [71]. Refeeding with a lower calorie provision and a slow energy increase may be a better approach for severely malnourished patients with chronic comorbidity, while higher caloric intakes might be reserved for moderately malnourished patients with acute illnesses [69]. Preventive supplementation with phosphate, potassium, magnesium, thiamine and other vitamins, trace elements, and minerals as well as sodium and fluid restriction are recommended too [66, 71]. Both meal-based approaches (with or without oral nutritional supplements) and combined approaches with nasogastric feeding can be used in inpatients requiring higher caloric intakes [69, 73]. Parenteral nutrition is not recommended unless no other form of refeeding is possible [69].

### Cancer

Up to 50–80% of patients with advanced cancer are at high risk of developing RFS [74], in particular individuals with head and neck cancer [75, 76]. Cancer cachexia cannot be arrested or reversed by any known form of nutritional, hormonal, or pharmacological treatment [77]. There are no specific guidelines on how to re-feed cancer patients at risk for RFS, being NICE recommendations [48] the most frequently used [29, 75, 76, 78, 79]. In patients eating little or nothing for more than 5 days, refeeding should be started with no more than 50% of the caloric requirements, with ≤ 10 kcal/kg/day in high-risk patients and ≤ 5 kcal/kg/day in very high-risk patients (BMI < 14 kg/m^2^ or negligible intake for 2 weeks or more) [48]. Owing to the potential benefit of protein intake on muscle anabolism, cancer patients should receive a protein intake of 1 g/kg/day up to 1.5 g/kg/day [79]. When oral refeeding is possible, the use of oral nutritional supplements can be useful in reaching nutritional goals [76]; if oral feeding is either impossible or insufficient, enteral, or parenteral nutrition should be considered [76], with slow progressive caloric increase to reach the full needs within 4–7 days [74]. In the case of cancer cachexia, a very cautious refeeding should begin by initially supplying about 25% of the estimated calorie requirement [77], with a very gradual caloric increase over several days, and a careful monitoring of phosphate and electrolytes serum levels [80].

## Conclusions

This narrative review provides the latest information on the management of RFS in light of the current evidence. Although RFS is a frequent condition that can have serious consequences above all in specific categories of inpatients, it is often undiagnosed and overlooked by physicians. Its knowledge is essential to avoid rapid and excessive nourishing of at-risk patients; thus, preventing serious complications, long hospital stays, and the increase in health costs.

## Electronic supplementary material

Below is the link to the electronic supplementary material.

Supplementary file1 (DOCX 14 kb)

## Data Availability

Not applicable.
