# Supplementary material for: The Refeeding Syndrome: a neglected but potentially serious condition for inpatients. A narrative review
Source: Intern Emerg Med. 2020 Oct 19;16(1):49–60. doi: 10.1007/s11739-020-02525-7 (PMC7843537; doi:10.1007/s11739-020-02525-7)
Supplement: Supplementary file 1 — Supplementary file1 (DOCX 14 kb) [file 11739_2020_2525_MOESM1_ESM.docx]

**Supplementary Table 1. Diseases and conditions predisposing to malnutrition [39]**

| **General category** | **Examples** |
| --- | --- |
| ***Predisposing to disease-related malnutrition with inflammation*** | |
| Chronic diseases leading to  catabolic inflammatory responses | cancer  chronic obstructive pulmonary disease (COPD) inflammatory bowel diseases  congestive heart failure  chronic kidney disease  other end-stage organ diseases  chronic inflammatory or infectious diseases  uncontrolled diabetes mellitus |
| Acute disease- or injury-related malnutrition | in-patients in an Intensive Care Unit with acute disease or trauma (e.g. major infections, burns, closed head injury)  major surgical procedures |
| ***Predisposing to disease-related malnutrition without inflammation*** | |
| Dysphagia | upper digestive obstruction  neurologic disorders (stroke, Parkinson's disease,  amyotrophic lateral sclerosis, dementia/cognitive  dysfunction) |
| Psychiatric diseases | anorexia nervosa, depression, chronic alcohol, or drug abuse |
| Intestinal malabsorption | short bowel syndrome |
| Advanced ageing | anorexia of ageing |
| ***Predisposing to malnutrition in the absence of diseases*** | |
| Hunger | Deprivation of food leading to prolonged fasting or severe caloric restriction for an extended period |
| Socioeconomic or psychologic related conditions | Poverty/social inequities  poor care  mourning  poor dentition  self-neglect  imprisonments  hunger strikers |
| Long-term poly-therapies | Anti-acids, diuretics, angiotensin-converting enzyme inhibitors, biguanides, opioids, protease inhibitors, chemotherapeutic and cancer medication, antihistamine, antibiotics, and antivirals |
